# Reply to: On the Clinical Utility of Hybrid Endoscopy in Crohn's Disease

**DOI:** 10.1002/deo2.70341

**Published:** 2026-04-28

**Authors:** Takahiro Ito

**Affiliations:** ^1^ Inflammatory Bowel Disease Center Sapporo Higashi Tokushukai Hospital Hokkaido Japan

To the Editor:

We thank Koulaouzidis and his colleagues [1] for their thoughtful engagement with our study on hybrid endoscopy in Crohn's disease [2] and welcome the opportunity to clarify several points.

The primary rationale for hybrid endoscopy is operational rather than preparatory. By combining small‐bowel capsule endoscopy (SBCE) and ileocolonoscopy on the same day, patients only need to undergo one bowel preparation and visit the hospital once instead of twice—a benefit reflected in the 82% preference for same‐day scheduling. In areas where panenteric capsule endoscopy is unavailable, this approach provides a practical method for assessing the entire intestinal tract and has the added capacity for targeted biopsy.

Regarding ileal cleansing, both groups had a median score of 2. The Mann–Whitney U test detected a distributional difference (*p* < 0.01), not a shift in medians. We did not intend to argue that more intensive purgation improves SBCE utility. Rather, we believe that the shortened small‐bowel transit time that occurs with purgative preparation is unlikely to compromise lesion detection because the concurrent improvement in ileal cleansing may compensate for the reduced observation time. We acknowledge that this hypothesis remains unproven and merits dedicated prospective investigation [3].

The central message of our study concerns treatment decision‐making. Among patients in clinical remission, approximately half had active endoscopic disease. Those without endoscopic remission who did not undergo treatment intensification had the highest relapse rates (*p* = 0.028). We agree that our Kaplan–Meier analysis is hypothesis‐generating and that seven de‐escalation cases preclude any firm conclusion on safety. Nevertheless, the signal favoring endoscopy‐guided intensification aligns with the treat‐to‐target paradigm [4] and supports objective monitoring beyond symptom control [5].

We acknowledge the limitations of a single‐center retrospective design. Multicenter prospective studies with standardized preparation, central image reading, and comparison with panenteric capsule endoscopy are required to confirm these findings.

## Author Contributions

Takahiro Ito drafted the manuscript.

## Funding

The author has nothing to report.

## Conflicts of Interest

The author declares no conflicts of interest.
